# Correction: Helium-induced damage in U_3_Si_5_ by first-principles studies

**DOI:** 10.1039/d1ra90139g

**Published:** 2021-08-24

**Authors:** Yibo Wang, Zhenbo Peng, Nianxiang Qiu, Heming He, Rongjian Pan, Lu Wu, Qing Huang, Shiyu Du

**Affiliations:** Engineering Laboratory of Nuclear Energy Materials, Ningbo Institute of Materials Technology and Engineering, Chinese Academy of Sciences Ningbo Zhejiang 315201 P. R. China qiunianxiang@nimte.ac.cn dushiyu@nimte.ac.cn; Institute of Energy Storage & Conversion Technology, Ningbo Polytechnic China; School of Mechanical and Electrical Engineering, Guangzhou University Guangzhou 510006 China; The First Sub-Institute, Nuclear Power Institute of China Chengdu Sichuan 610005 China

## Abstract

Correction for ‘Helium-induced damage in U_3_Si_5_ by first-principles studies’ by Yibo Wang *et al.*, *RSC Adv.*, 2021, **11**, 26920–26927. DOI: 10.1039/D1RA04031F.

The authors regret that an incorrect version of Fig. 4 was included in the original article. The correct version of Fig. 4 is presented below.

**Fig. 4:**
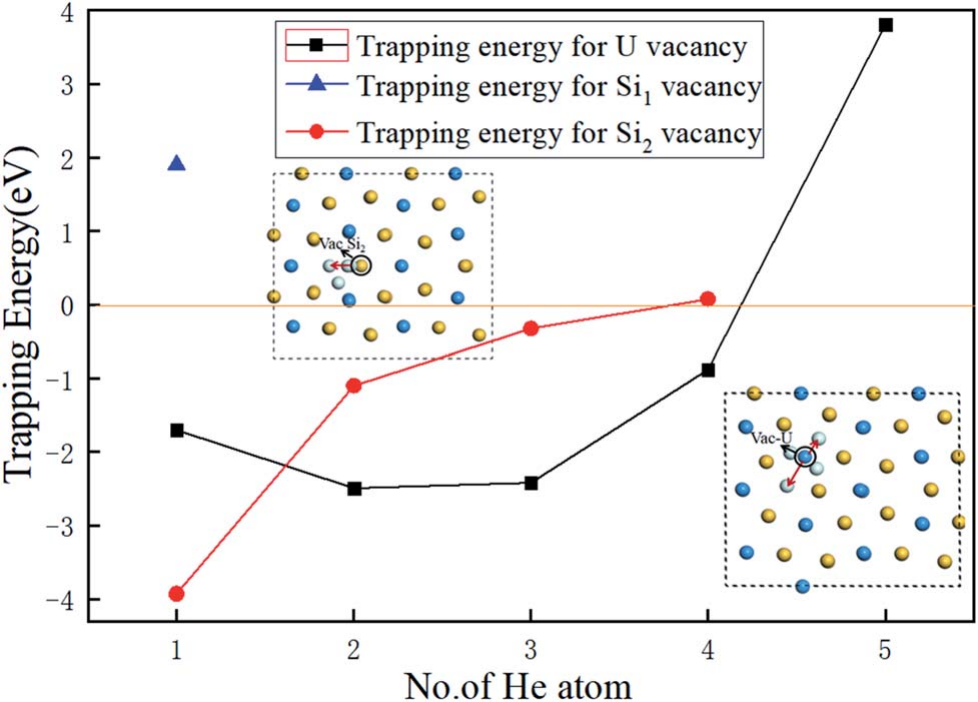
The dependence of the trapping energy on the number of He atoms trapped in Vac-U, Vac-Si_1_, and Vac-Si_2_ vacancies of U_3_Si_5_.

The Royal Society of Chemistry apologises for these errors and any consequent inconvenience to authors and readers.

## Supplementary Material

